# Patients’ experiences of attending emergency departments where primary care services are located: qualitative findings from patient and clinician interviews from a realist evaluation

**DOI:** 10.1186/s12873-021-00562-9

**Published:** 2022-01-22

**Authors:** Delyth Price, Michelle Edwards, Freya Davies, Alison Cooper, Joy McFadzean, Andrew Carson-Stevens, Matthew Cooke, Jeremy Dale, Bridie Angela Evans, Barbara Harrington, Julie Hepburn, Aloysius Niroshan Siriwardena, Helen Snooks, Adrian Edwards

**Affiliations:** 1 PRIME Centre Wales, Cardiff University School of Medicine, Cardiff, Wales; 2 Division of Population Medicine, Cardiff University School of Medicine, Cardiff, Wales; 3 Warwick Medical School, University of Warwick, Coventry, UK; 4 Swansea University Medical School, Swansea University, Swansea, Wales; 5 School of Health and Social Care, University of Lincoln, Lincoln, UK

**Keywords:** Patient experience, Emergency department, Realist evaluation, Primary care services, Qualitative

## Abstract

**Background:**

Patient experience is an important outcome and indicator of healthcare quality, and patient reported experiences are key to improving quality of care. While patient experience in emergency departments (EDs) has been reported in research, there is limited evidence about patients’ specific experiences with primary care services located in or alongside EDs. We aim to identify theories about patient experience and acceptability of being streamed to a primary care clinician in an ED.

**Methods:**

Using theories from a rapid realist review as a basis, we interviewed 24 patients and 106 staff members to generate updated theories about patient experience and acceptability of streaming to primary care services in EDs. Feedback from 56 stakeholders, including clinicians, policymakers and patient and public members, as well as observations at 13 EDs, also contributed to the development of these theories, which we present as a programme theory.

**Results:**

We found that patients had no expectations or preferences for which type of clinician they were seen by, and generally found being streamed to a primary care clinician in the ED acceptable. Clinicians and patients reported that patients generally found primary care streaming acceptable if they felt their complaint was dealt with suitably, in a timely manner, and when clinicians clearly communicated the need for investigations, and how these contributed to decision-making and treatment plans.

**Conclusions:**

From our findings, we have developed a programme theory to demonstrate that service providers can expect that patients will be generally satisfied with their experience of being streamed to, and seen by, primary care clinicians working in these services. Service providers should consider the potential advantages and disadvantages of implementing primary care services at their ED. If primary care services are implemented, clear communication is needed between staff and patients, and patient feedback should be sought.

## Background

Patient experience is an important outcome of healthcare quality [1], and patient-reported experiences are key to improving the quality of care [2]. Increasing attention is also being paid to patient experience and satisfaction as *indicators* of healthcare quality, as both measures can identify shortcomings, improve health care quality, and promote patients’ choice and voice [3–5]. While ‘experience’ generally relates to patients’ memories of what happened during their care, ‘satisfaction’ is more closely related to patients’ opinions and feelings about what happened in relation to their expectations [5]. Differentiating between these two terms, but ultimately focussing on gaining insight into both, is important in healthcare research. Exploring patient experience enables researchers to evaluate outcomes for patients, while exploring patient satisfaction can provide insights into the gap between expectation and actual experience [5].

In this article, we discuss patients’ positive and negative experiences as ‘experience’, and use the term ‘satisfaction’ when these reported experiences are related to patients’ prior expectations.

Patients’ negative experiences of emergency care have been attributed to waiting times, overcrowding, inadequate communication, a lack of privacy and uncomfortable ED environments [6–13]. Sonis et al. describe a conceptual “logic model” for patient experience in EDs [12, 13]. Key themes contributing to patients’ experiences were staff-patient communication, waiting time, and staff empathy and compassion [12, 13]. Patients’ preferences for which type of treating clinician they see have been reported as ‘neutral’, as low visibility of primary care services and/or a higher level of integration with the ED team means that patients often do not distinguish between being treated in a primary care service within an ED, or in an ED itself [14]. While patients are aware of significant pressures on the NHS and are reported as seeing value in different types of clinicians (such as general practitioners) working in or alongside EDs [15], there is limited evidence about patients’ specific experiences with primary care services located in or alongside EDs.

Increasing pressure on EDs, including a perceived increase in attendance of those with “primary care problems” and staffing challenges, has led to interest in different models of service delivery, such as the use of general practitioners (GPs) or primary care services in or alongside EDs [25]. In our realist review of the impact of GPs working in or alongside EDs, we developed initial theories relating to patient experience in EDs (see appendix 1) using data from nine papers [16]. Data used to develop theories came from both UK and international (US, Canada, Netherlands) studies, using a range of methodological approaches including qualitative, randomised, cross-sectional and mixed-methods, with sample sizes ranging from 102 to 4684 patients [14, 17–24]. Studies used to develop our initial theories evaluated a range of services and models, including comparisons between walk-in clinics, community primary care services and EDs; EDs with co-located walk-in centres; GP led walk-in centres; and EDs with integrated primary care clinicians [14, 17–24].

However, data to support theories about patient experience and satisfaction were limited. In the next phase of our work (described here) we collected qualitative data to further test and develop these theories. We sought to understand the experiences of patients attending EDs with different primary care models [25], focussing on acceptability to patients of being streamed to a primary care clinician, and patients’ experiences of seeing a primary care clinician or ED clinician in these different models. Our objectives were to identify how these findings could be used to guide service development, improve care quality and patient experience, and to develop a programme theory of transferable lessons.

## Methods

### Wider study

Our NIHR funded realist study ‘*Evaluating effectiveness, safety, patient experience and system implications of different models of using GPs in or alongside Emergency Departments*’ (HS&DR Project 15/145/04) was commissioned to explore the impact of changes in service delivery.

Realist methodology is a form of theory-driven evaluation, which asks what works, for whom, under what circumstances, and how [26]. Realist methodology is well-suited to the evaluation of complex systems such as healthcare because it allows for deep insights into problems to be gained, and possible solutions to them to be proposed [26, 27].

To develop initial theories, we used a rapid realist review [16] to identify mechanisms (M) which explain how or why certain contexts (C) relate to outcomes (O), generating ‘context-mechanism-outcome’ (CMO) configurations [28] regarding patient experience of being seen by a primary care clinician working in or alongside an ED. These CMO configurations were then developed into initial theories, which informed our interview schedules. In undertaking this work, we followed the RAMESES standards (see appendix 3).

### Data collection and samples

#### Sites

A national survey of all type 1 EDs (consultant-led EDs, open 24-h with full resuscitation facilities) in England and Wales provided the basis for our site selection [16]. We conducted follow-up interviews with Clinical Directors at 21 sites [28]. Thirteen sites were then purposively selected for case-study, based on several variables to ensure they included three different models of emergency department primary care services (“inside-integrated”, “inside-parallel” and “outside-onsite”) (see Table 1), no primary care service (controls), and varying contexts such as size, geographical location, and levels of attendance [25].

**Table 1:** Primary care service models

Primary care service model	Description
Inside-integrated	A primary care service fully integrated with the emergency medicine service, where staff see both primary and emergency care patients (n= 3).
Inside-parallel	A separate primary care service within the emergency department, for patients with primary care type problems (n= 4).
Outside-onsite	Primary care service is elsewhere on the hospital site (n = 3).
Control site	No model of using GPs in ED (n = 3)

#### Patients

We conducted semi-structured telephone interviews with 24 patients/ carers of patients who visited the ED for one of five conditions which could potentially be managed by a primary care clinician. These - chest pain, cough and breathlessness, abdominal pain, back pain, and fever in a child under 10 years old – were identified using literature on ambulatory sensitive care conditions [29–39] and views of our stakeholder group [25]. Patients were purposively sampled (based on this range of ‘marker conditions’ and from different EDs and service models) and were contacted via post within three months of their visit to the ED, to inform them of their eligibility to take part in the study and request their consent for an interview. Interviews were conducted by one researcher (ME). Interviews topics included patients’ reasons for attending the ED, what their expectations were before visiting the ED, and how satisfied they were with waiting time, tests and investigations, treatments, and the general level of care provided by their clinician (see appendix 4 for interview topic guide).

We aimed to interview 60–120 patients, but experienced several recruitment challenges, such as limited face-to-face interaction between researchers and patients on site, and limited availability of research nurses to assist with patient recruitment at some sites [40]. However, our sample includes patients across a range of ages (including parents of children) with various conditions, seen by both primary care clinicians and ED clinicians, in the different service models included in our study (see appendix 2 for list of all patients).

#### Staff

We conducted semi-structured interviews with 106 staff members including Clinical Directors, GPs, ED clinicians and nurses working in the selected 13 EDs. Interviews with Clinical Directors were conducted by telephone in advance of site visits, and other staff interviews were conducted on-site by two researchers (ME and AC) during visits, or via telephone following the visits where there was limited availability. The realist teacher-learner interview technique was used, whereby initial theories were presented to participants to explore how different mechanisms and contexts may result in both intended and unintended outcomes [41]. Due to our low patient recruitment, staff interviews were used to provide additional perspectives on patient experience and acceptability of streaming, as staff regularly receive second-hand information and feedback on patients’ experiences in EDs.

#### Observations

Observations of ED reception and streaming/triage assessments were conducted by two researchers at the 13 sites, over the one- to three-day visits (range of day / evening, weekday / weekend shifts). Observations of how the different systems worked, supplemented by informal opportunistic conversations with a wide range of staff, provided additional staff perspectives on patients’ experiences and acceptability of being streamed to a primary care clinician.

### Data analysis and stakeholder engagement

During data collection, we conducted a preliminary analysis of our interview and observation data and, using our initial theories as a basis, generated further theories to present to stakeholders. Our patient and public involvement (PPI) co-applicants assisted with this preliminary data analysis, to provide a patient and public perspective. We then held a stakeholder conference with 56 stakeholders including ED clinicians, general practitioners, service managers, policymakers and patient and public contributors, at which we presented these initial theories on patient satisfaction and acceptability of primary care streaming from our preliminary analysis [25]. Our PPI co-applicants co-led a workshop where stakeholders worked in mixed groups to discuss each theory, provide feedback, and suggest additional contexts and mechanisms for consideration. Feedback from stakeholders led to further refinement of our theories, which then guided the subsequent analyses.

The patient and staff interview and observation data were then analysed by two researchers (ME and DP), in QSR NVivo 12, based on these new themes. Two researchers (ME and DP) coded the data independently and then reviewed each other’s work to reach agreements before developing context, mechanism, outcome configurations [28]. Theories were then generated regarding how different mechanisms (e.g. how patients are streamed in an ED) explain how or why certain contexts (e.g. why patients attend an ED) relate to outcomes (e.g. a positive experience, or acceptability of primary care streaming).

**Fig. 1:**
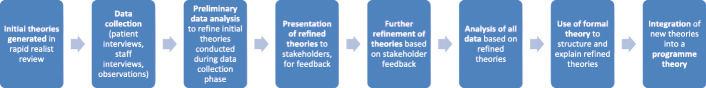
Data collection, analysis, and theory generation process

We then aimed to integrate these theories as a ‘Programme Theory’ [42] to explain why using primary care clinicians in or alongside EDs may or may not work to improve experience or acceptability of streaming, for whom, and in what specific circumstances. A programme theory is an overall high-level theory summarising how the intervention works, developed using the theories refined from the data [16]. See Fig. 1 for diagram of the data colletion, data analysis and theory refinement process.

### Patient and public involvement

Two patient and public contributors are co-applicants in our study (BH, JH), and are involved in overseeing and delivering the study with other co-applicants, as research management group members. They were involved in analysing our patient data, recruiting public contributors to our stakeholder conference, and delivering workshops to stakeholders. A group of 13 public contributors attended our stakeholder conference to provide insight and feedback into our theories regarding patient experience and acceptability of being streamed to a primary care clinician in an ED [25]. We supported all public contributors involved in this study, in line with best practice [43].

## Results

We now present our qualitative data to support, refute, or refine our initial theories, and identify new theories [26]. Data from patient interviews (*n* = 24), staff interviews (*n* = 106), observations (*n* = 23), and feedback from 56 stakeholders have contributed to these theories about: acceptability of being streamed to a primary care clinician and patients experience and satisfaction relating to waiting times and investigations.

### Acceptability of being streamed to a primary care clinician

Our earlier literature review did not generate any theories specific to the acceptability of being streamed to a primary care clinician [16], so new theories were developed based on patients’ reported views on the acceptability of being streamed (patient-derived theories), and clinicians’ perceptions of acceptability to patients of being streamed or redirected (clinician-derived theories).

#### Patient-derived theory: acceptability of being streamed to a primary care clinician

Some patients described having difficulty accessing or obtaining satisfactory care from their community primary care service, and believed the ED was the right place to attend to receive comprehensive assessment, diagnosis, and treatment on the same day. Patients generally found it acceptable to be streamed to a primary care clinician in the ED.

*“I just needed somebody to give me sort of help... where it come from it didn’t really matter ... Getting to the hospital, you’ve obviously come to the right place … and I wasn’t getting any advice at all off my local GP” [Patient with cough & breathlessness seen by GP at hospital 13]*

*New theory: A patient may present to the ED with a problem for which they are finding it* ***difficult to access care*** *or for which they have been* ***dissatisfied with the care received from their community primary care service*** *(C). If they have* ***no expectations of which type of clinician they should be seen by at the hospital****,* ***trust the initial assessment process****, and believe that they will get good advice at the hospital (M), then they will find the process of being streamed to a GP* ***acceptable*** *(O).*

#### Clinician-derived theory: streaming to a non-visible primary care service

Clinicians reported that when patients are not aware that there is a primary care service at the ED, but an explanation is given about why they are being streamed to a primary care clinician, they seem to find this acceptable.

Interview data showed techniques of managing patient expectations, and therefore improving the acceptability to patients of being streamed to a primary care clinician, included reassuring patients of the experience, knowledge and seniority of primary care clinicians:

*“There’s odd ones that will say “I’ve been to the GP, I’ve come here to get....” and then you say oh well … I can get a GP to see you, they don’t normally complain because I do say it’s a very experienced doctor, we’ve also got Junior doctors, and you’re in the same building, if there’s anything needed you’ll be getting it, and I reassure the patients.” [Emergency Nurse Practitioner at hospital 4]*

*New theory: If a patient is* ***streamed to a GP*** *(C) and an ED nurse* ***explains*** *that this is because the GP is the* ***most appropriate clinician*** *with the best knowledge and experience to deal with their complaint (M), the* ***patient will be aware*** *that they are seeing a GP, and why, and may* ***trust the initial assessment process*** *(M), and therefore find being streamed to a GP* ***acceptable*** *(O).*

#### Clinician-derived theory: apparent acceptability to patients of being re-directed to a community primary care service

We found during fieldwork observations that redirection after an initial assessment was common during a triage or streaming assessment. We were unable to interview patients who had been redirected to other services, as this was outside the scope of our study, however clinicians reported on mechanisms that they thought influenced the acceptability to patients of being redirected to separate services in different primary care models.

In the three EDs included in our study that had procedures in place to redirect primary care patients to booked appointments in community primary care services, clinicians reported that this removes a potentially long wait in ED for patients to see a primary care clinician. One staff member reported that patients appear to find this acceptable, because they can go home and will be seen by their own community primary care service the same day, and are therefore more likely to feel that they are being helped, rather than sent away to look for care elsewhere. The acceptability to patients of redirection to community primary care was also influenced by having a guaranteed same-day appointment at their community primary care service, without a long wait in the ED.

*The person working as a navigator said that it generally doesn’t feel that people are inconvenienced by being sent back to their local service because they are going back to the area that they live in, and have actually come out of their area to get to the ED. [Field notes by ME, hospital 4]*

*New theory: If patients arrive at the ED because they* ***want to be seen*** *by a clinician (C) and they are* ***assessed in ED and redirected*** *to a booked same-day appointment in their community primary care service or at an out-of-hours (OOH) GP service (M), they* ***avoid having to wait*** *in the ED (M), and* ***may find being streamed to other primary care services acceptable*** *(O).*

At two hospitals, patients were streamed to the primary care service for a triage assessment and could then be redirected to a community primary care service. However, this meant they were assessed twice before being redirected, taking additional time and causing frustrations for patients. At hospital 11, a GP explained how patients waited to be seen before being redirected.

*“They don’t really discharge anybody. If it’s not for them they send it here, as their default even if it’s something completely that we’re not going to help them with, they still send them here, they wait three hours and then they get told we can’t help you which is not great”. [GP at hospital 11]*

*New theory: When patients attend ED with a* ***problem*** *which could be dealt with in primary care and are streamed to a primary care service (C)* ***which triages and then redirects them to another service****, leading to multiple* ***waiting and assessments*** *(M), they may find the streaming and redirection process* ***unacceptable*** *(O).*

#### Stakeholder conference feedback

Our stakeholder group provided feedback on additional contexts and mechanisms for each of our theories, which are summarised in Table 2 below.

**Table 2:** Stakeholder feedback on theories of patients’ acceptability of streaming (3rd November 2019)

Theory origin	Theory	Stakeholder feedback
Patient-derived	*A patient may present to the ED with a* ***persistent problem*** *for which they are finding it* ***difficult to access care*** *or for which they have been* ***dissatisfied with the care received*** *from their community primary care service (C). If they have* ***no expectations of who they should be seen by at a hospital, trust the initial assessment process, and believe that they will get good advice at a hospital*** *(M), then they may find the process of being streamed to a GP* ***acceptable*** *(O).*	• Patients are usually happy if their expectations are met. • Timely access is important - patients just want to see a doctor - from their point of view they get to see a doctor the same day. • Acceptability depends on how unwell the patient feels and how worried they are about their health. • Sometimes a GP referral when patient wants a second opinion is less acceptable to the patient. • Patient assumptions that ‘better’ advice is received in ED than GP – not necessarily true. • A good GP can be better than lots of investigations.
Clinician-derived	*If a patient is* ***streamed to a GP*** *because they are the* ***most appropriate clinician*** *with the best knowledge and experience for the complaint(C) and an ED nurse* ***explains*** *this reasoning to the patient, the* ***patient will be aware*** *that they are seeing a GP and may* ***trust the initial assessment process*** *(M), therefore finding being streamed to a GP* ***acceptable*** *(O).*	• Patients are more concerned with timeliness rather than who they see. • Communication is a key mechanism here. Important factors: speedy, appropriate, knowledgeable. • Might depend on patient’s condition and why they have gone to ED. • Depends on how ill the patient feels or how worried they are.
Clinician-derived	*Patients who are assessed in an ED primary care service model* ***and streamed or redirected to a booked appointment*** *with their community primary care service or hospital-based OOH GP service (C) will* ***not have to wait in the ED to be seen and can be seen locally that day****, or go home and return for an appointment at the OOH primary care service later (M), so may find being steamed to other primary care services* ***acceptable*** *(O).*	• Might be time consuming, some patients may not find being sent away acceptable • Safety concerns if patients do not attend the community primary care service. • Depends on how unwell they feel. • What is the wait to get an appointment? Still faster than a GP appointment? • Depends on whether they wanted/expected to see a GP. • How far away they live, availability of parking. • Has a good explanation been given?
Clinician-derived	*When patients with a* ***non-urgent care problem*** *which could be dealt with in primary care present to an outside-onsite service model (C)* ***which redirects them to their community GP service****, leading to them* ***waiting and being seen twice*** *before being sent away (M), they may find the streaming and redirection process* ***not acceptable*** *(O).*	• ‘Unacceptable’ depends on severity of symptoms. • Patients may be left feeling they should not have gone to ED. • How they are treated is important, patients need reassurance that it is okay to go home. • Quality of communication is important; an outcome that has an appointment is okay. 2 stops is not good, 1 stop is ideal – patient might feel ‘fobbed off’.

### Patients’ experience and satisfaction relating to waiting time and investigations

Through patient interviews, we found evidence to support some of the initial theories in our rapid realist review (see appendix 1), which we have refined to reflect nuances in context and mechanisms.

#### Waiting time

*Initial rough theory: patients who attend ED (C), and are seen by a* primary care clinician*, may experience shorter waiting times and fewer investigations to treat their complaint (M), leading to increased satisfaction with the experience (O).*

From patient interviews, we found that some patients expected a long wait to be seen and were satisfied when their wait was shorter because they had seen a primary care clinician. Patients did not express levels of satisfaction regarding the number of investigations received, but did express satisfaction with the amount of time spent at the ED.

*“I was very surprised actually to be in and out as quickly as we were. I was expecting a good sort of three or four hours waiting around in A&E and for people to assess and then re-assess. So actually, I think the fact that we were probably in and out within sort of maybe an hour and a half was really good.” [patient with cough & breathlessness seen by GP at hospital 7]*

We refined our initial theory to include patient expectations as part of the context, and have removed investigations from the mechanism because we did not find that this was reported as contributing to waiting time satisfaction.

*New theory: when patients* ***expect a long waiting time*** *in the ED (C), and they are* ***seen more quickly than they expected by a primary care clinician*** *in ED, and sent home (M), may be* ***satisfied*** *with their experience (O).*

#### Investigations

*Initial theory: patients who attend ED having already seen their community primary care clinician and not received the level of investigation or treatment they expected (C), and are seen by a primary care clinician in the ED, may once again not receive the level of investigation or treatment they expected (M) and therefore be dissatisfied with their experience (O).*

One of our initial theories related to patients being dissatisfied if they did not receive the level of investigations they expected in the ED. We were not able to collect data to explore this further. However, we did find examples of patients who were seen by a GP and were satisfied when they felt that they had received appropriate investigations, and the results were explained to them during their visit.

*“...they looked at the x-ray and said there are no fractures, and explained probably why it was very painful, and they then said that the results from the ECG were normal. They didn’t send me away worrying about what the results were.” [patient with cough & breathlessness seen by GP at hospital 3]*

*New theory: if a patient attends the ED* ***expecting specific investigations and treatment*** *(C), and they receive investigations which* ***provide insight into their condition****, then they may feel that they have received* ***appropriate care*** *in the ED (M), and be* ***satisfied with their experience*** *(O).*

## Discussion

### Main findings

We found that patients from our sample had no expectations or preferences relating to which type of clinician they were seen by, as there was a general expectation that they would be appropriately assessed and treated at an ED. Patients with one of five selected conditions therefore found being streamed to a primary care clinician in the ED acceptable. Clinicians felt that patients found it acceptable to be streamed to either an on-site OOH primary care service, or redirected to an off-site community primary care service, if there was a clear explanation and no long wait in ED, and they were redirected after an initial timely assessment.

We found that patients reported a positive experience when they felt their presenting complaint had been appropriately dealt with and they had been seen in a timely manner. Patients also felt positive about their experience when clinicians explained the need for investigations and how these contributed to clinical decision-making and treatment plans.

### Strengths & limitations

Recruitment challenges mean our sample of patients from which our findings are drawn is limited [40]. While our sample of patients had a range of conditions and ages, our limited eligibility criteria (e.g., specific conditions) may have restricted the representativeness and size of our sample, and future studies may wish to consider widening eligibility criteria for such research [40]. However, we were able to mitigate this small sample by gaining clinicians’ views on patient experience, supplemented by observations in the EDs and additional feedback from a large group of stakeholders, including 13 patient and public members. We used rigorous realist methods to add to the limited body of evidence about specific service models, identifying new mechanisms that are associated with ED use where primary care services are located. Importantly, we have not had access to quantitative data on patient experience, such as local patient-reported outcome or experience measures, but our wider realist evaluation of the impact of implementing primary care services in or alongside ED on patient safety, experience, effectiveness, and resourcing will include quantitative evaluations of attendances, re-attendances, admissions and use of investigations.

### Context of other literature & programme theory

Our findings are consistent with other reviews and conceptual models of patient experience [6, 13] and extend these findings to include the additional context of primary care service models at EDs, and the experiences of patients with conditions suitable for primary care who seek urgent care at an ED. Our findings highlight the importance of short waiting times and clear communication for patients, which is consistent with other studies [6–13]. In line with previous research, we found that some patients were not aware that they had been seen by a primary care clinician when they were interviewed, particularly if the primary care service was not ‘visible’ as a distinct service [14]. Overall, we found little variation in experience or satisfaction between patients who were seen by primary care clinicians or ED clinicians, in line with theories on patient satisfaction reported in our realist review [16].

The most frequent theme from Sonis et al.’s “logic model” [12] of ED patient experience was ‘staff-patient communication’, followed by ‘wait time’ and ‘staff empathy and compassion’, which resonates with the mechanisms we have described in our theories of patient experience. Other contexts and features identified in that review were consistent with findings from our interviews with staff members, including ED crowding and ED environment, staff medical competence, staff communication, staff experience, ED leadership and policy factors [12].

Other themes which are consistent with the contexts described in our theories were patient experience of pain and discomfort (which motivated them to seek urgent care at an ED), and condition acuity and triage.

**Fig. 2:**
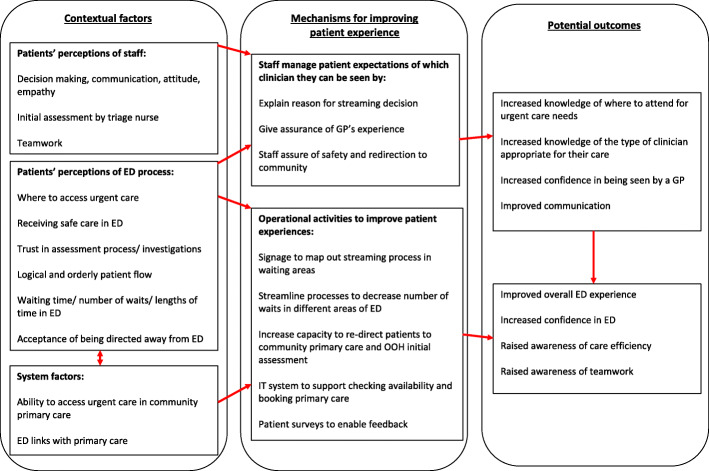
Programme theory of patients’ experiences of streaming and redirection to primary care services

We used this conceptual model as a framework for developing a programme theory (see Fig. 2). We have mapped out contextual factors (system factors and patients’ prior experience / expectation factors) and mechanisms for staff to manage patients’ experience and operationally manage the service to influence outcomes (patients’ acceptability and satisfaction).

## Conclusion

Our findings suggest patients attending EDs with urgent care needs report a positive experience of being streamed to on-site primary care services, or redirected to off-site community care services. This occurs if the reasons for streaming are explained to them, they are informed about necessary investigations, they feel safely managed and have a shorter wait. We outline priority concepts on which service providers implementing or improving primary care services in their EDs could focus, based on what matters to patients. Notably, the need for regular measurement of patient experience to empirically inform decision-making about the design and redesign of services will be a key component of such continuous quality improvement efforts and in the pursuit of achieving value from service delivery.

### Implications for policy and practice

The mechanisms identified as contributing to patient experience, satisfaction, and acceptability of being streamed to a primary care clinician in ED can help guide service development and quality improvement. Stakeholder consultation provided us with further mechanisms to consider, such as availability of transport, ensuring patients feel safe, and clear communication. We recommend the following:

i) Consider consequences of implementing primary care services at an ED

Service providers should consider the potential advantages and disadvantages of implementing primary care services in their ED, in relation to their specific contexts. We have shown that patients generally have positive experiences when they are seen by a primary care clinician working in or alongside an ED, or by an ED clinician in a service where there are primary care clinicians working. Multiple streaming and redirection pathways to primary care services (at an ED, OOH service on the hospital site, or off-site community services) can be advantageous, and the effectiveness of pathways being signposted in waiting areas to inform patients and manage expectations should be explored.

ii) Seek patient feedback

Service user feedback should be sought to evaluate service changes and provide insights into which mechanisms make primary care streaming acceptable to patients. Patients’ evaluations of the quality of information and explanation when being streamed/redirected could also prove useful for such service evaluation. Regular patient feedback on streaming processes, explanations, and experiences of being seen by a primary care clinician at the ED should be sought to allow monitoring and improvement of services, including for staff training.

iii) Ensure clear communication

Clear communication with patients, to inform them of why and to whom they are being streamed, providing clear explanations about whether investigations in the ED are available and necessary, and the role of investigations in clarifying diagnoses and treatment plans, are key to enhancing patient experience, satisfaction, and acceptability of being streamed.

### Further research

Our findings have informed a programme theory about patients’ experiences of streaming and redirection to primary care services. This requires further testing, in wider settings and service models, as a basis for interventions or innovations to improve the service. Any future interventions which arise from these or subsequent findings will require further evaluation of whether they do improve patient experience of attending EDs where primary care services are implemented, or other outcomes such as patient flow and safety.

## Data Availability

The datasets used and/or analysed during the current study are available from the corresponding author on reasonable request.

## Appendix 1

### Initial theories of patient experience from Rapid Realist Review

Theory 1:

Patients who attend ED having had difficulty accessing their usual GP personally, or where there is a problem with access to local primary care (C), and are seen by a GP and feel that their complaint has been dealt with appropriately (M), report a satisfied experience (O).

Theory 2:

Patients who attend ED and are seen by a GP may not perceive any difference in the care they received (or may not be aware they were seen by a GP) (M), and so will report no difference in satisfaction with their treatment (O).

Theory 3:

Patients who attend ED having already seen their GP and not received the level of investigation or treatment they expected (C), and are seen by a GP may once again not receive the level of investigation or treatment they expected (M) and are therefore dissatisfied with the experience (O).

## Appendix 2

**Table 3:** Patient recruitment log

Patient no.	Condition	Treating clinician	Hospital site	Model
1	Cough & breathlessness	ED clinician	GPED03	Inside integrated
2	Cough & breathlessness	ED clinician	GPED04	Inside parallel
3	Cough & breathlessness	ED clinician	GPED06	Inside parallel
4	Cough & breathlessness	GP	GPED03	Inside integrated
5	Cough & breathlessness	GP	GPED05	*No model**
6	Cough & breathlessness	GP	GPED06	Inside parallel
7	Cough & breathlessness	GP	GPED09	Inside parallel
8	Cough & breathlessness	GP	GPED10	Outside onsite
9	Cough & breathlessness	GP	GPED13	Outside onsite
10	Back pain	ED clinician	GPED05	*No model**
11	Back pain	ED clinician	GPED06	Inside parallel
12	Back pain	GP	GPED03	Inside integrated
13	Back pain	GP	GPED04	Inside parallel
14	Back pain	GP	GPED11	Outside onsite
15	Abdominal pain	ED clinician	GPED14	Inside integrated
16	Abdominal pain	ED clinician	GPED14	Inside integrated
17	Abdominal pain	GP	GPED03	Inside integrated
18	Abdominal pain	GP	GPED06	Inside parallel
19	Child with fever	ED clinician	GPED04	Inside parallel
20	Child with fever	ED clinician	GPED04	Inside parallel
21	Child with fever	GP	GPED13	Outside onsite
22	Chest pain	ED clinician	GPED02	Control
23	Chest pain	ED clinician	GPED10	Outside onsite
24	Chest pain	ED clinician	GPED11	Outside onsite

** site ruled out as case study site, patient interviews still included in sample*

## Appendix 3

### RAMESES standards checklist

1. Title identified as realist review
2. Abstracts should ideally contain brief details of the study’s background, review question or objectives; search strategy; methods of selection, appraisal, analysis and synthesis of sources; main results; and implications for practice.
3. Explain why the review is needed and yes what it is likely to contribute to existing understanding of the topic area.
4. State the objective(s) of the review and/or the review question(s). Define and provide a rationale for the focus of the review.
5. Any changes made to the review that Yes was initially planned should be briefly described and justified.
6. Explain why realist synthesis was considered the most appropriate method to use.
7. Describe and justify the initial process of exploratory scoping of the literature
8. State and provide a rationale for how the iterative searching was done. Provide details on all the sources accessed for information in the synthesis. For example, where electronic databases have been searched, details should include, for example, the name of the database, search terms, dates of coverage and date last searched. If individuals familiar with the relevant literature and/or topic area were contacted, indicate how they were identified and selected.
9. Explain how judgements were made Yes about including and excluding data from documents, and justify these.
10. Describe and explain which data or information were extracted from the included documents and justify this selection.
11. Describe the analysis and synthesis processes in detail. This section should include information on the constructs analyzed and describe the analytic process.
12. Provide details on the number of documents assessed for eligibility and included in the review with reasons for exclusion at each stage, as well as an indication of their source of origin (for example, from searching databases, reference lists and so on).
13. Provide information on the characteristics of the documents included in the synthesis.
14. Present the key findings with a specific focus on theory building and testing.
15. Summarize the main findings, taking into account the synthesis’ objective(s), research question(s), focus and intended audience(s).
16. Discuss both the strengths of the review and its limitations. These should include (but need not be restricted to) (a) consideration of all the steps in the synthesis process and (b) comment on the overall strength of evidence supporting the explanatory insights that emerged. The limitations identified may point to areas where further work is needed.
17. Where applicable, compare and contrast the synthesis’ findings with the existing literature (for example, other reviews) on the same topic.
18. List the main implications of the findings and place these in the context of other relevant literature. If appropriate, offer recommendations for policy and practice.
19. Provide details of funding source (if any) for the synthesis, the role played by the funder (if any) and any conflicts of interests of the reviewers.

## Appendix 4

**Table 4:** Patient Interview Guide

**Experience/reasoning for visiting the ED**	• I’m interested on the day that you went to the emergency department, can you explain to me how you were feeling that day and what led you to seek help in the emergency department? • How long had you been experiencing symptoms? • Did you seek any help from any other health service for the problem (111/ooh GP/ local GP)? • What made you decide to come to the emergency department when you did, was it the amount of pain you were in, locality of hospital, urgency of treatment?
**Expectations/Satisfaction**	• Have you had much experience of going to an emergency department before? • What kind of service did you expect when you went to the ED? (waiting time, tests to be done, treatment) • Did you have any expectations or any preferences about who you might be seen by? • Did you know that there were GPs working in the emergency department? • How satisfied (or not) would you say you are with the care you received by the GP (or ED Staff)? You waited … to be seen and left at … .how do you feel about the length of time you were waiting?
**Investigations/treatment**	• If had tests - You had … …. (test), how did you feel about having to have this done? • If no investigations – did you expect to get some tests done that were not done? If yes, how did you feel about that? • The treatment you received was … … .were you happy with that? • Would recommend the service to the friends and family … … … …
**Follow up/ contact with health services after leaving ED**	• The follow up advice you were given was … … … ..were you happy with that? • What happened after you left the ED, did you need to go and see a GP in the community?
**GP in ED vs GP in community**	• You were seen by a GP and GPs are skilled in dealing with the problem that you have, would it have been a possibility for you to have visited your own GP for the problem? Would it have been easier or more difficult? (longer to access the service and get treated) • What is it like trying to see a GP in the community? • Did you know that you were going to be seen by a GP (or that you had been seen by a GP)? • The GP gave you medication (similar to what a GP might have done in your surgery). What do you think might have happened if you had rang your GP surgery (or GP OOH) for the same problem? Do you think you would have got similar treatment in a similar time-frame?
**Future intentions**	• Where would you go to seek treatment if you had the same problem again? Would you come to the emergency department again? What would be the main reason that would influence your decision on where to go?
**Views on GP services in ED**	• What are your views on where GPs should be working? Do you think it is useful to have them in the emergency department? • Some people might say that if they know there are GPs working in the emergency department it might be easier to see them there than go to a local surgery? What do you think about that? • Do you think there might ever be a case of more people going to the emergency department to get seen by a GP?

## Abbreviations

C: Context
M: Mechanism
O: Outcome
CMO: Context, mechanism, outcome
ED: Emergency Department
GP: General Practitioner
OOH: Out of hours
